# Sensors for the detection of ammonia as a potential biomarker for health screening

**DOI:** 10.1038/s41598-021-86686-1

**Published:** 2021-03-30

**Authors:** Peter P. Ricci, Otto J. Gregory

**Affiliations:** Department of Chemical Engineering, University of Rhode Island, East Alumni Avenue Suite 360, Kingston, RI 02881 USA

**Keywords:** Chemical engineering, Sensors and biosensors

## Abstract

The presence of ammonia within the body has long been linked to complications stemming from the liver, kidneys, and stomach. These complications can be the result of serious conditions such as chronic kidney disease (CKD), peptic ulcers, and recently COVID-19. Limited liver and kidney function leads to increased blood urea nitrogen (BUN) within the body resulting in elevated levels of ammonia in the mouth, nose, and skin. Similarly, peptic ulcers, commonly from *H. pylori*, result in ammonia production from urea within the stomach. The presence of these biomarkers enables a potential screening protocol to be considered for frequent, non-invasive monitoring of these conditions. Unfortunately, detection of ammonia in these mediums is rather challenging due to relatively small concentrations and an abundance of interferents. Currently, there are no options available for non-invasive screening of these conditions continuously and in real-time. Here we demonstrate the selective detection of ammonia using a vapor phase thermodynamic sensing platform capable of being employed as part of a health screening protocol. The results show that our detection system has the remarkable ability to selectively detect trace levels of ammonia in the vapor phase using a single catalyst. Additionally, detection was demonstrated in the presence of interferents such as carbon dioxide (CO_2_) and acetone common in human breath. These results show that our thermodynamic sensors are well suited to selectively detect ammonia at levels that could potentially be useful for health screening applications.

## Introduction

Chronic kidney disease (CKD) is a condition characterized by the decrease or limitation of kidney function. In 2015, 44.6 million people were diagnosed with CKD with many reported over the age of 60^1^. Unfortunately, due to the escalation of COVID-19, the overall number of patients suffering from limited liver and kidney function has increased. COVID-19 is an infectious disease caused by severe acute respiratory syndrome coronavirus 2 (SARS-CoV-2). This disease has many known symptoms including fever, cough, and shortness of breath. However, recent research has confirmed significant variations in the concentration of numerous biochemicals in patients with COVID-19^2–8^. Those suffering from serious COVID-19 symptoms have shown increased concentrations of blood urea nitrogen (BUN), due to the effects of kidney damage^2–8^. Studies show that COVID-19 patients displayed signs of kidney dysfunction, including proteinuria (59%) and hematuria (44%)^4^.

BUN is a measure of the amount of nitrogen in your blood due to the presence of urea. Urea, a waste biochemical, can build up as a result of kidney damage. This build-up of urea affects liver function and thus, slows the processing of ammonia^9^. Increases in blood urea nitrogen suggest that increased concentrations of ammonia in the body may be quantifiable. Other studies have shown that concentrations of blood urea nitrogen can be correlated by 95% to concentrations of ammonia in the breath^10^. Similar to COVID-19, many studies have reported increases in BUN and ammonia levels in patients with CKD^11–13^. However, increased ammonia levels have also been displayed in patients with H. *pylori*^14,15^, a bacterium which increases the production of ammonia in the stomach^16^. Thus, ammonia has the potential to be a detection biomarker for liver and kidney screening as well as monitoring for H. *pylori* infection.

As mentioned above, limited liver and kidney function as well as H. *pylori* infection can lead to elevated levels of ammonia in the body and eventually in the mouth, nose, and skin**.** Unfortunately, detection of ammonia in the nose and on the skin is rather challenging due to the relatively small concentrations exhibited in these locations (< 50 parts-per-billion (ppb) in the nose and < 10 ppb on the skin)^17^. However, significant ammonia levels have been reported in the mouth and subsequently in breath. Typical breath ammonia levels can range between 29 and 688 ppb with average of 265ppb^18^. These levels are substantially increased in stage 3–5 CKD which range from 1392 to 3660 ppb respectively. The presence of ammonia at these levels provides the impetus for a potential detection system capable of non-invasive health screening^19^.

Several platforms exist that are capable of detecting ammonia in the vapor phase at very low concentrations. However, each has their own set of drawbacks in terms of detecting ammonia efficiently and effectively. For example, fluorescence chromatography techniques have been investigated in an attempt to detect ammonia at the 100 ppb level^20^. However, the ammonia fluorene shows some overlap with various background gases, such as nitrogen and thus, would require extended calibration for most applications. Detection of ammonia has also been achieved through other analytical techniques including spectroscopy^21–23^, ion mobility spectrometry^24–26^, and gas chromatography^27^. Unfortunately, these techniques possess a relatively large footprint relative to small-scale chemical sensors. Other researchers have been able to successfully detect ammonia in the vapor phase using a variety of quartz crystal microbalance^28^, metal oxide based^29,30^ sensor platforms and selective sensor arrays^31,32^. These techniques display impressive sensitivity at trace levels but exhibit only minimal selectivity in the presence of interferents such as volatile organic compounds (VOCs) and humidity.

Our thermodynamic sensor platform^33–38^ utilizes microheaters deposited onto ultrathin yttria-stabilized-zirconia (YSZ) ribbons that are coated with a metal oxide catalyst for the detection of vapor phase compounds. The response of the sensor is somewhat orthogonal in that it measures two different heat effects simultaneously. The first heat effect is produced almost instantaneously as the analyte molecule (i.e. ammonia) is catalytically decomposed into known decomposition products upon interaction with the catalyst surface. The decomposition products then interact with the metal oxide catalyst resulting in selective redox reactions and thus a second heat effect. Both heat effects release energy causing a change in electrical power required to maintain the sensor temperature setpoint. These changes are measured and recorded using feedback/control circuitry. In general, oxidation reactions are known to release heat producing negative responses as the electrical power required to maintain the sensor temperature is decreased. Conversely, reduction reactions absorb heat and produce positive responses due to an increase in required electrical power.

## Results and discussion

**Figure 1:**
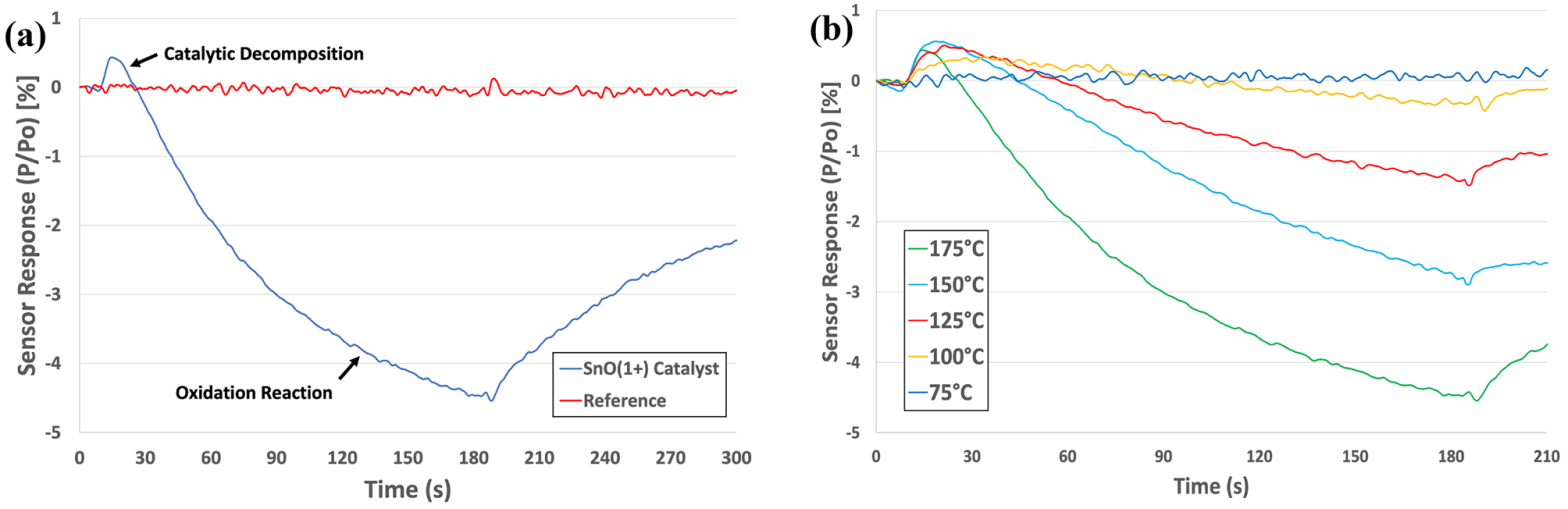
Response of a thermodynamic sensor to 5 ppm of ammonia. The sensor employed a SnO^1+^ catalyst (**a**) Response of a thermodynamic sensor employing a SnO^1+^ catalyst to 5 ppm ammonia at a variety of temperatures (**b**).

Figure 1a shows the response of a thermodynamic sensor employing a tin oxide (SnO^1+^) catalyst to 5 parts-per-million (ppm) ammonia operating at 175 °C. The SnO^1+^ catalyst displayed exceptional sensor response (~ 4.5%) to ammonia due to the subsequent oxidation reaction from its decomposition products. Recently, we have shown that highly porous metal oxide films resulted in greater catalytic activity and improved sensor response^38^. When porous SnO^1+^ catalyst films were employed for the detection of ammonia, greater catalyst surface area improved sensor response. The reference sensor here did not employ a catalyst coating and instead was used to mitigate hydrodynamic effects such as changes in flowrate and pressure as well as heat capacity differences. During normal operation, the reference signal is subtracted from the active catalyst signal such that only the catalytic effect is measured. Figure 1a shows that the reference sensor was unresponsive to the ammonia vapor as expected.

Our detection mechanism is unique in that it monitors two different heats effects, simultaneously. An initial heat effect due to the release of energy via catalytic decomposition, which is followed by a secondary heat effect due to redox reactions between the decomposition products and the metal oxide catalyst. In general, the redox reactions produce heat effects that far exceed those associated with catalytic decomposition in magnitude and thus, dominate the overall sensor response. However, in the case of ammonia, the decomposition energy is relatively large depending on the catalyst (> 200 kJ/mol) enabling the catalytic decomposition heat effects to be readily measured. The heat effect produced by the catalytic decomposition of NH_3_ in the presence of SnO^1+^ is endothermic^39^ in nature and thus, produces a positive response (~ 1.2%). When the decomposition products interact with the catalyst a large exothermic heat effect was observed due to redox reactions (Fig. 1a). Thus, the response to ammonia is very unique, due to its relatively large decomposition energy, making the detection of ammonia in highly selective.

In an effort to make our sensor platform more portable than other sensor platforms, the detection of ammonia was investigated at substantially lower operating temperatures as shown in Fig. 1b. Here, the detection of 5 ppm ammonia was achieved at temperatures as low as 100 °C. In general, the operating temperature of the sensor plays a key role in the detection process. Traditionally, for tin oxide (SnO^1+^), higher operating temperatures (> 100 °C) favor the occurrence of redox reactions which dominate the sensor response and produce large heat effects. At relatively low operating temperatures (< 100 °C), catalytic decomposition is more prevalent as the oxidation state of SnO^1+^ is unaffected by the presence of the decomposition products. In most cases, the two heat effects are opposite in sign, which adds a built-in redundancy to the platform, making orthogonal detection using a single catalyst possible. In the case of ammonia, the reduction in operating temperature has a large effect on the redox reaction but has almost no effect on the catalytic decomposition at temperatures above 100 °C. The detection of ammonia at lower temperatures, greatly reduces the power consumption; e.g. at 175 °C, the sensor requires ~ 400mW to maintain the desired temperature. However, at 100 °C, the sensor requires only 250mW, which is optimal for a portable device. These YSZ-based sensors can cool quickly to room temperature (on the order of seconds) after deactivation, thus, allowing for a rapid overall duty cycle. Combined with < 1% variation in signal strength, this sensor platform can detect ammonia at trace levels in real-time without any delay needed for the sensor to recover to baseline.

**Figure 2:**
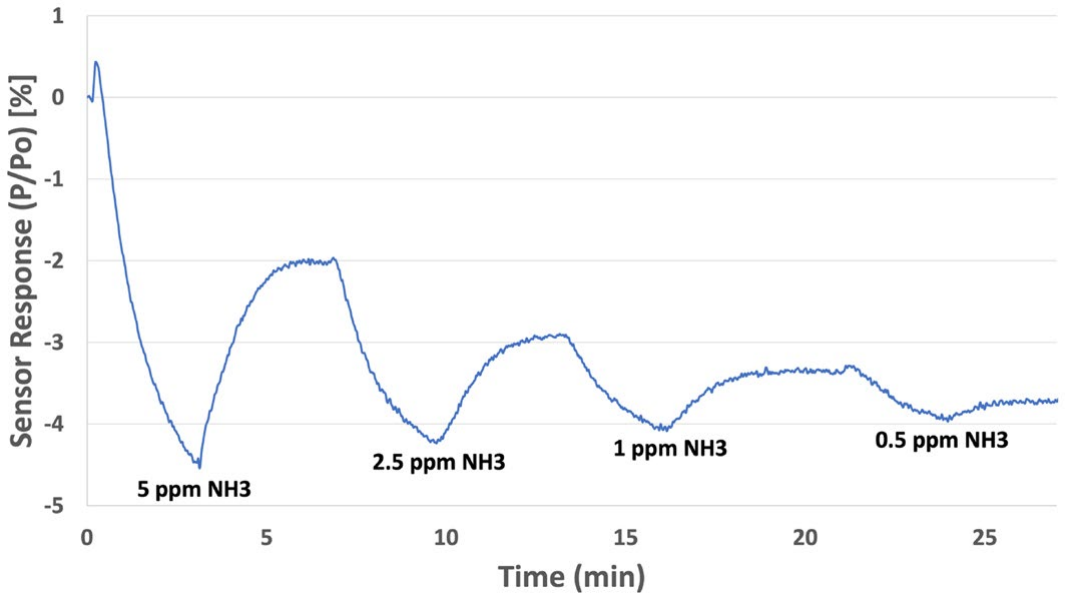
Response of a thermodynamic sensor employing SnO^1+^ catalyst to ammonia. Concentrations ranged from 0.5 to 5 ppm.

Based on the selectively of the detection mechanism and the relatively low-power requirements exhibited by our sensor platform, our sensor is uniquely suited for a variety of potential screening applications. As mentioned above, ammonia concentrations in breath greatly exceed those exhibited from the nose and skin. In any effort to test the early viability of our sensor for screening applications, early experiments using ‘breath-like’ conditions were simulated. Based on the initial concentration of ammonia in our specialty gas (5 ppm), further dilution was necessary using air to obtain ppb concentrations. Figure 2 shows sensor response to ammonia at concentrations ranging from 5 to 0.5 ppm (or 500 ppb). These concentrations coincide well with the range of ammonia concentrations exhibited by patients with stage 3–5 CKD (1.4 to 3.7 ppm) as described above.

**Figure 3:**
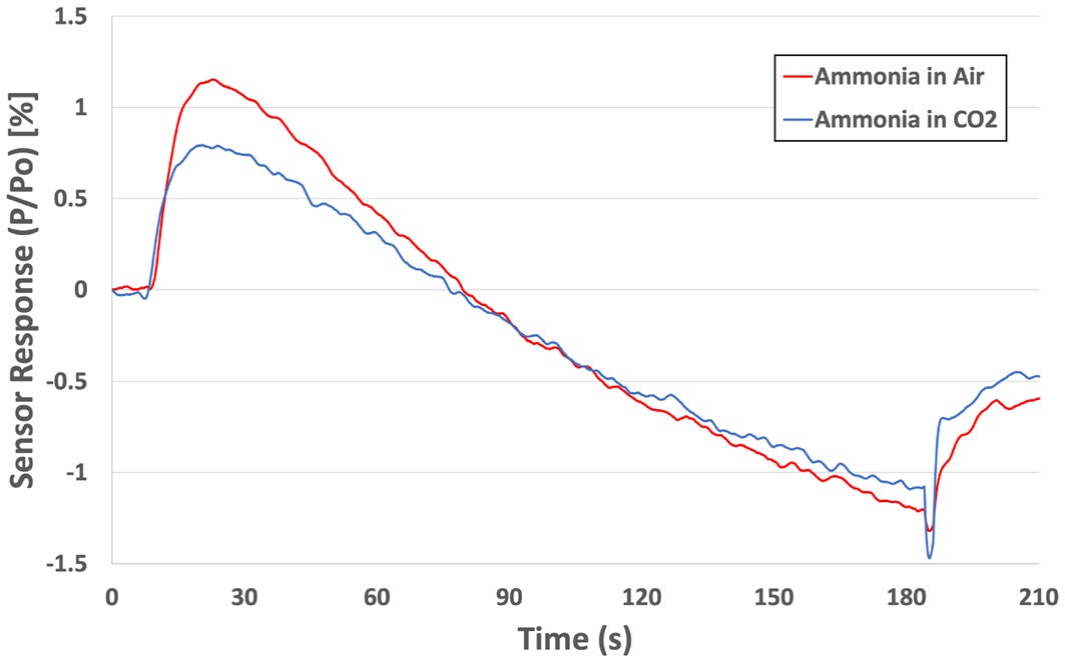
Responses of thermodynamic sensor to 5 ppm of ammonia in air and CO_2_.

**Figure 4:**
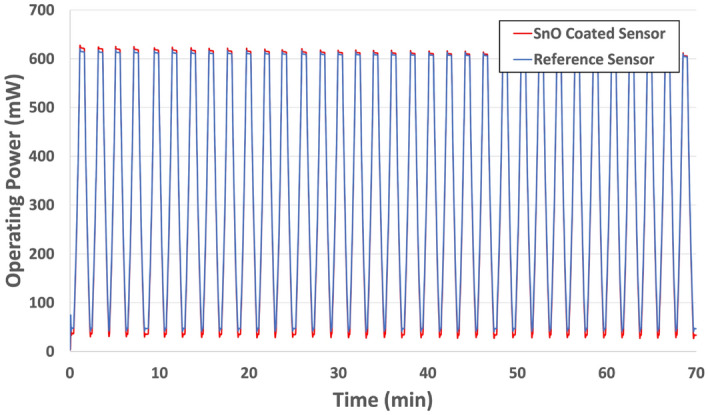
Thermal cycling data (~ 35 cycles) for the SnO^1+^ catalyst sensor and the reference sensor in air (100% RH).

In addition to a unique sensor response, our thermodynamic sensor platform also has remarkable selectivity for ammonia. In a potential breath screening application, selective identification of ammonia among an extensive library of interferents is required. In addition to humidity, exhaled breath is composed primarily of air with varying concentrations of over 100 VOCs^32^. However, carbon dioxide (CO_2_) is considered the most abundant VOC in breath (4–5% by concentration)^40^. In order to mimic this environment, our thermodynamic sensor was exposed to ammonia diluted with CO_2_ to a concentration of 1 ppm (Fig. 3). The results show that the redox response of our sensor was largely unaffected by the presence of the CO_2_ and displayed the same response to ammonia as that in pure air (~ 1%). Interestingly enough, the unique catalytic decomposition response is shown to decrease slightly (~ 1.2 to 0.8%). This result was expected due to the increased concentration of CO_2_ which increased the oxidation potential in the testing atmosphere and thus, diminished the reduction reaction produced by catalytic decomposition. In addition to CO_2_, humidity is also a known interferent in exhaled breath. Our sensor and SnO^1+^ catalyst have displayed impressive time of life with the ability to operate for hundreds of cycles in most environments including 100% relative humidity (RH). Figure 4 shows a portion (~ 35 cycles) of the sensor’s thermal cycling stability. Here, the sensors were cycled from 25 °C to 250 °C where the power required to heat the sensors to 250 °C is ~ 615mW. After such extensive cycling, a nitrogen regeneration process was developed to regenerate the oxide catalyst without having to replace the sensor. Here, pure nitrogen was passed over a heated sensor for 15 min to activate catalytic sites.

**Figure 5:**
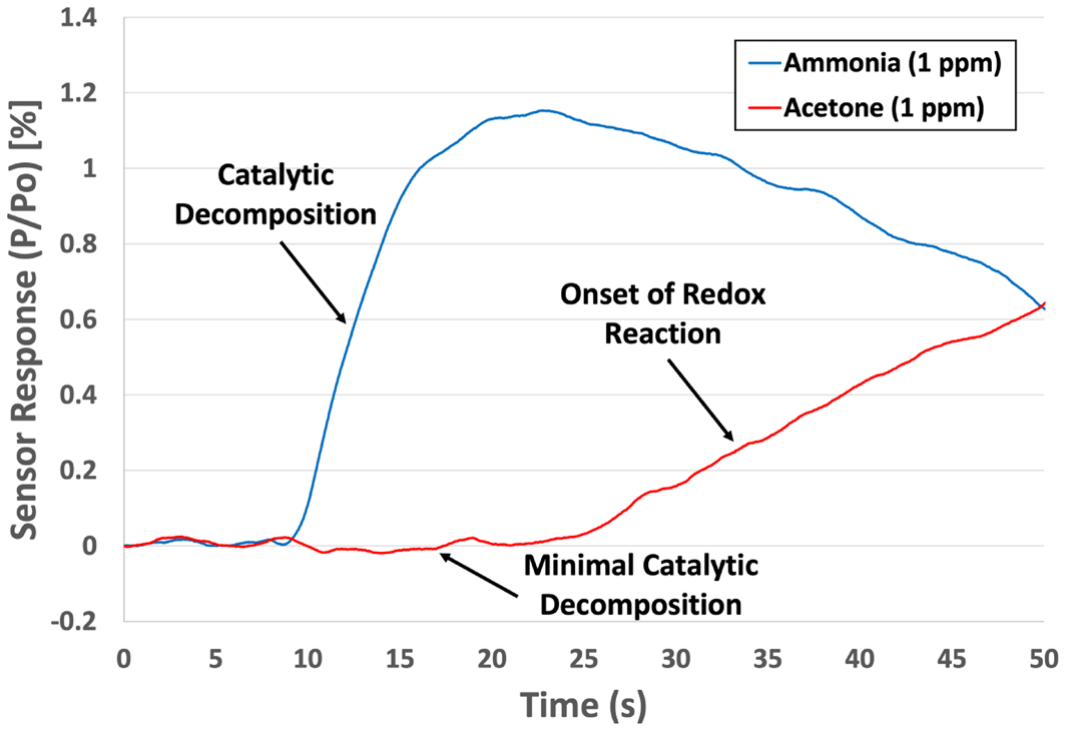
Responses of a thermodynamic sensor to 1 ppm ammonia and 1 ppm acetone.

In addition to CO_2_ and humidity, detection of ammonia in the presence of other high vapor pressure exhaled breath VOCs is essential. These VOCs exhibit different chemical compositions and vapor pressures that can make selective detection of a single molecule increasingly challenging. For our experiment, one VOC was introduced to the sensor simultaneously with ammonia. Figure 5 shows the response of our thermodynamic sensor to 1 ppm ammonia and 1 ppm acetone. Acetone is considered a breath biomarker for a number of other biological processes and possesses a relatively high vapor pressure (0.3 atm). Due to its low decomposition energy (< 100 kJ/mol), minimal heat effects associated with catalytic decomposition were expected while largely endothermic heat effects were anticipated from the redox reactions. Typically, these types of responses can interfere with the detection of other target molecules. However, ammonia’s catalytic decomposition generates a response before the redox reactions occur and thus, can be detected in the presence of these high vapor pressure compounds like acetone. Between 10 and 25 s, the sensor exhibited a highly selective catalytic decomposition response to ammonia while remaining unresponsive to acetone (Fig. 5). This difference in signals demonstrated the selectivity of ammonia over that of acetone and thus, a unique “fingerprint” was produced that could be used for identification purposes. Also, detection of ammonia within this timeframe, means the rapid identification of ammonia in the presence of the other hundreds of VOC molecules could be possible.

Using our ultrasensitive, thermodynamic sensor, the detection of ammonia in ‘breath-like’ conditions was demonstrated in real time. Specifically, ammonia was detected at the ppb level, in part, due to its relatively high decomposition energy. The sensor displayed a highly unique and selective response in which both an endothermic catalytic decomposition and exothermic oxidation reaction were observed. The detection of 5 ppm ammonia was achieved at temperatures as low as 100 °C, resulting in a substantial reduction in power (250mW from 400mW), which allows for improved portability of the sensor platform. In addition, the thermodynamic sensor displayed unparalleled selectivity. We were able to detect ammonia in the presence of potential breath interferents such as CO_2_ and acetone. These VOCs, combined with high levels of RH, make detection of ammonia in exhaled breath relatively difficult for any detection system. However, due to the highly sensitive and selective response of our thermodynamic sensors to ammonia, we believe that our sensor platform could be well suited to detect ammonia in a variety of health screening applications.

## Methods

### Sensor fabrication

**Figure 6:**
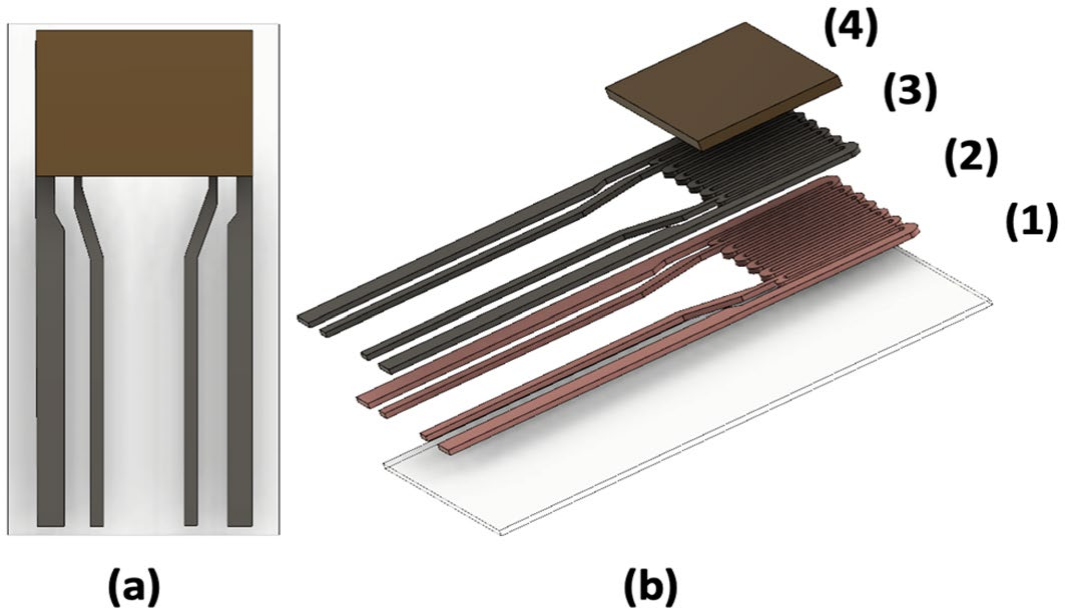
Top view of the thermodynamic sensor (**a**). Cutaway view of the thermodynamic sensor (**b**) showing the various layers comprising the sensor: YSZ substrate (1), copper adhesion layer (2), palladium microheater (3), and metal oxide catalyst (4).

The components of the thermodynamic sensor were fabricated on ultrathin (20 µm) yttria-stabilized zirconia (YSZ) ribbons measuring 1.6 cm × 0.7 cm. Photolithography techniques were used to pattern palladium (1 µm thick) microheaters on these ultrathin substrates. Prior to deposition of the palladium films, a 400 Å thick layer of copper was sputter-deposited onto the substrate to act as an adhesion layer between the microheater and the YSZ. All sputtering was done using an MRC 8667 sputtering machine. Each microheater has four thin film leads, which allow communication between the sensor and the digital control system. A schematic of the various layers comprising the thermodynamic sensor is shown in Fig. 6. Since the noble metals, Pd and Pt, exhibited poor adhesion to the YSZ, a variety of metals including Ni, Cr, Cu, and Ti were investigated as adhesion promoters. Ultimately, Cu was chosen for this purpose due to its affinity for yttria in the YSZ. The thermodynamic sensors employed a variety of metal oxide catalysts including SnO^1+^, CuO, ZnO, FeO, and In_2_O_3_. However, SnO^1+^ was the catalyst used throughout this study due to its highly selective response to ammonia. The catalyst layer was sputter deposited as a continuous film over the surface of the microheater serpentine (Fig. 6).

### Testing procedure

**Figure 7:**
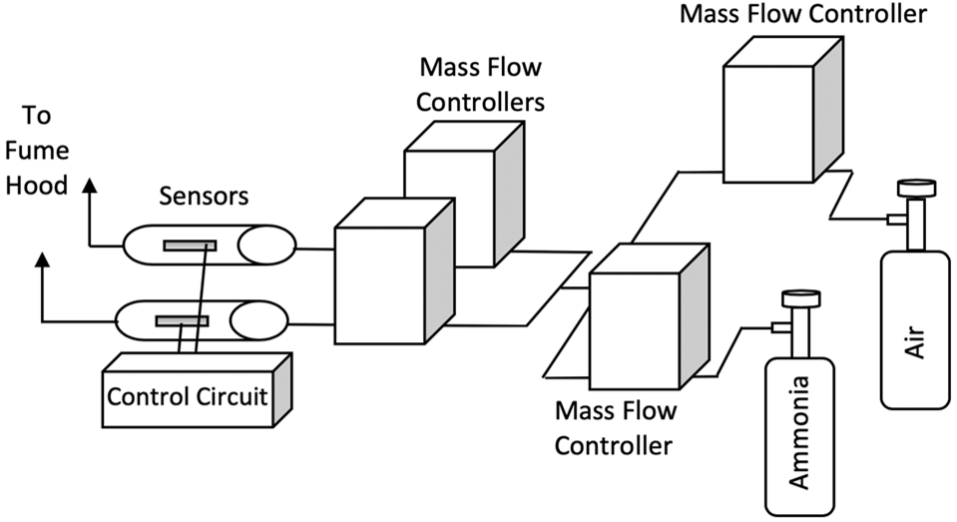
Schematic of apparatus used for the detection of ammonia.

As previously described by Ricci et al.^38^, a digital control system was used to supply electrical power to the microheaters using proprietary software. The set point temperature was determined from the temperature coefficient of resistance (TCR) and resting resistance of the palladium microheaters at room temperature. The flow of air from a pressurized gas cylinder was divided into two identical streams, each of which was delivered to the catalyst-coated microheater and reference microheater using mass flow controllers (Allicat Scientific Mass Flow Controllers with Flow vision software). The mass flow controllers provided a stable flowrate of 200 SCCM for all experiments in this study. The microheater sensors were then heated to a predetermined set point temperature, and a power/resistance baseline was established^33–38^. At the start of a run, the gas flow was simultaneously switched from dry air to ammonia and delivered downstream to the sensors. A schematic of the test bed is shown in Fig. 7. The ammonia dilution limit of our testing apparatus is currently 0.5 ppm as shown above. However enhancements are expected to deliver significantly lower concentrations in the future.

Our thermodynamic sensor platform consists of two separate microheaters: one coated with a metal oxide catalyst, and the other left uncoated, which acts as a reference^33–38^ The reference is used to differentiate between heat effects that arise from catalyst-analyte interactions and those due to hydrodynamic effects including sensible heat effects. By subtracting the reference signal from the catalyst-coated signal, the heat effect due to catalytic decomposition alone is measured. These decomposition reactions can be either exothermic or endothermic and require a change in electrical power to keep the temperature of the two microheaters constant.

As these heat effects develop, the software maintains a constant temperature set point by either adding or subtracting electrical power to the catalyst-coated microheater. These electrical power changes required to maintain a constant temperature is in effect, the thermodynamic heat effect associated with these analyte-catalyst interactions^33–38^. The measured power difference is recorded using proprietary software and divided by baseline power; i.e. the power required to maintain the desired temperature setpoint. Here, the change in electrical power is reported as a percentage. A typical experiment is run at temperatures as high as 175 °C and requires several minutes to complete, since the sensors were allowed to reach peak response before the analyte supply was shut off. The peak response is the power required to maintain the desired temperature setpoint and reach equilibrium and depends on the response to a specific analyte.
